# Spatiotemporal patterns and meteorological associations of mosquito density: insights from mosquito surveillance in Chengdu, China

**DOI:** 10.3389/fcimb.2026.1948618

**Published:** 2026-09-16

**Authors:** Shuwei Pei, Wei Zhang, Dan Kuang, Chunxia Luo, Wenjia Tian, Shuangfeng Fan

**Affiliations:** 1 Affiliation of Disinfection and Vector Control, Chengdu Center for Disease Control and Prevention (Chengdu Institute of Health Supervision), Chengdu, China; 2 Policy Research Office of the CPC Chengdu Pidu District Committee, Chengdu, China

**Keywords:** lagged associations, machine learning, meteorological drivers, mosquito density, nonlinear modeling

## Abstract

**Objectives:**

This study aimed to investigate the spatiotemporal distribution of mosquito density and its associations with meteorological factors in Chengdu, China, and to compare the predictive performance of different models, thereby providing a scientific basis for mosquito-borne disease surveillance and early warning.

**Methods:**

Mosquito surveillance data were collected from 22 county-level administrative areas in Chengdu between 2023 and 2025, with meteorological data obtained from the Chengdu Meteorological Bureau. The Wilcoxon signed-rank test was used to compare mosquito density between urban and rural habitats, while NMDS, PERMANOVA, and SIMPER analyses were performed to evaluate differences in mosquito community structure. A generalized additive model (GAM) was applied to evaluate nonlinear responses of mosquito density to meteorological factors. Using an 80:20 split grouped by collection date, we compared GAM, random forest (RF), and extreme gradient boosting (XGBoost) performance and characterized temperature–sunshine joint responses.

**Results:**

Mosquito density showed a unimodal seasonal pattern from 2023 to 2025, peaking in June or July. Higher densities were observed in peripheral areas (e.g., Chongzhou, Dujiangyan, and Qionglai), whereas central urban areas showed lower densities. *Culex tritaeniorhynchus* and *Culex pipiens pallens* were the two dominant. Mosquito density was significantly higher in rural than urban habitats (*P* < 0.001), and mosquito community structure differed significantly between urban and rural habitats (*P* = 0.001). GAM revealed significant nonlinear associations of mean daily temperature and cumulative sunshine duration over lag days 1–7 with mosquito density (both *P* < 0.001). Mosquito density increased significantly between 15.59 and 22.44 °C and reached an estimated peak at 25.02 °C. Model comparisons showed that RF achieved the highest predictive performance, followed by XGBoost.

**Conclusion:**

Mosquito density in Chengdu exhibits significant spatiotemporal heterogeneity, with temperature and sunshine duration identified as key meteorological factors. Machine learning models showed superior predictive performance over GAM. To prevent and control mosquito-borne diseases, meteorological factors should be incorporated into the existing monitoring system.

## 1 Introduction

Mosquitoes are among the most important arthropod vectors worldwide and are capable of transmitting a wide range of pathogens, including *Plasmodium* spp., Japanese encephalitis virus, dengue virus, chikungunya virus, and Ross River virus (Ferraguti, 2024). Globally, vector-borne diseases cause more than 700,000 deaths annually, with mosquito-borne diseases (MBDs) accounting for a substantial proportion of the disease burden (Wei et al., 2026; World Health Organization, 2024). In recent years, global climate change, accelerated urbanization, and land-use changes have facilitated the geographical expansion of mosquitoes and altered their seasonal activity patterns, posing new challenges to the prevention and control of MBDs (de Souza and Weaver, 2024). Between 2005 and 2024, more than 410,000 cases of MBDs and over 2,000 associated deaths were reported in China, mainly in southern and southwestern regions (Liu et al., 2025). Sichuan Province, located in southwestern China, experiences a warm and humid climate with seasonal rainfall, providing favorable conditions for mosquito breeding. Historically, Sichuan was a malaria-endemic region and continues to face the risk of imported cases (Gong et al., 2023). Moreover, as one of the high-incidence regions for Japanese encephalitis in southwestern China, Sichuan Province has consistently reported incidence rates above the national average (Liu et al., 2018; Du et al., 2025). Following the first locally transmitted dengue cases reported in 2019, 641 dengue cases were recorded in Sichuan Province during 2019–2024, affecting 119 districts/counties across 20 prefecture-level cities (Yue et al., 2021; Cao et al., 2025). These findings underscore the growing risk of MBDs in the region and highlight the need for enhanced mosquito surveillance and transmission risk prediction.

Mosquito density is an important entomological indicator for assessing vector population dynamics, activity levels, and potential disease transmission risk (Wang et al., 2025). Compared with case-based surveillance data, mosquito density monitoring can more rapidly capture abnormal changes in vector populations and activity, facilitating the early identification of potential transmission risks before disease outbreaks occur (Li et al., 2019). Mosquito distribution in urban environments is highly heterogeneous. Different areas vary in urbanization levels, land-use types, and microclimate conditions, which can collectively influence mosquito breeding sites, adult resting habitats, and host availability (Perrin et al., 2022; Schierer et al., 2025). Therefore, characterizing the spatial heterogeneity of mosquito density and identifying the environmental factors associated with it at the district and county levels are essential for mosquito risk assessment and targeted vector control.

Mosquitoes, as typical climate-sensitive organisms, are significantly influenced by meteorological conditions. Temperature regulates mosquito development rates, survival, and blood-feeding behavior, whereas precipitation affects reproduction by altering the availability and persistence of aquatic breeding habitats (Mordecai et al., 2019; Bastos et al., 2024). However, the relationship between meteorological factors and mosquito density is often nonlinear. Previous studies have shown that moderate increases in temperature and precipitation may promote mosquito development and population growth, whereas excessively high temperatures or heavy rainfall may reduce mosquito density by increasing physiological stress or disrupting larval breeding habitats (Lim et al., 2021; Mancuso et al., 2023). Because the mosquito life cycle comprises multiple developmental stages, including eggs, larvae, pupae, and adults, the effects of meteorological factors on adult mosquito density are often manifested only after these developmental processes have occurred, resulting in temporal lag effects (Prasad et al., 2024). In addition, different meteorological factors may also interact to jointly regulate mosquito population dynamics. For example, sunshine duration may modify mosquito responses to temperature by influencing local thermal conditions (Lee et al., 2024; Meuti, 2025). Beyond their effects on mosquito density, climatic factors can also shape the transmission dynamics of mosquito-borne infections, with local climatic conditions contributing to seasonal variation in dengue and malaria incidence (Cazelles et al., 2023). Therefore, comprehensively considering the complex response patterns of mosquito populations to meteorological factors is essential for accurately understanding mosquito population dynamics and improving risk prediction.

In this study, mosquito surveillance data collected in Chengdu, Sichuan Province, China, between 2023 and 2025, were used to characterize mosquito density, community composition, and spatial distribution patterns. A generalized additive model (GAM) was applied to characterize nonlinear responses of mosquito density to meteorological factors, while GAM, RF, and XGBoost models were compared in terms of predictive performance and their estimated joint temperature–sunshine response patterns. The findings are expected to provide foundational data for effectively controlling the mosquito population and MBDs in the region.

## 2 Materials and methods

### 2.1 Study sites and mosquito surveillance

This study was conducted from 2023 to 2025 across 22 county-administrative areas of Chengdu, Sichuan Province, China, including municipal districts, county-level cities, counties, the Chengdu High-tech Industrial Development Zone, and the directly administered area of Sichuan Tianfu New Area. Chengdu is located in southwestern China and covers approximately 14,335 km². The municipality includes a densely urbanized central plain and more agricultural and semi-natural landscapes toward the periphery. Mosquito surveillance was conducted twice per month, during the first and second halves of each month, from April to November each year. Surveillance was performed at 99 fixed sampling sites that were revisited twice monthly during each surveillance season throughout the study period, comprising 66 urban sites (22 residential areas, 22 parks, and 22 hospitals) and 33 rural sites (19 farms and 14 pasture sheds). Residential areas, parks, and hospitals were classified as urban habitats based on their location in built environments with frequent human activity, whereas farms and pasture sheds were classified as rural habitats based on their association with agricultural production and livestock farming. Therefore, the dataset followed a repeated-measures design, with temporally linked observations collected from the same sampling locations over time. Mosquitoes were collected using light traps. The traps were activated 1 h before sunset and operated continuously until 1 h after sunrise on the following day. Trained field workers inspected the traps and recorded the abundance of captured mosquitoes. All specimens were transported to the laboratory, classified by sex, and morphologically identified to the species level under a dissecting microscope using the Classification and Identification of Important Medical Insects of China (Lu and Wu, 2003). Identification was based on scutal and abdominal scaling patterns, wing venation and scales, leg banding, and palpal and proboscis morphology. The “Other” category comprised uncommon identified taxa that were not analyzed separately, specimens identifiable only to the genus level, and damaged specimens lacking sufficient diagnostic characters. Mosquito density was calculated as the number of female mosquitoes captured per trap-night.

### 2.2 Meteorological data

Meteorological data were obtained from the Chengdu Meteorological Bureau, covering the entire study period. The dataset included mean temperature (°C), mean relative humidity (%), rainfall (mm), mean wind speed (m/s), and sunshine duration (h). All meteorological variables were acquired at a daily resolution and temporally matched with mosquito surveillance dates to ensure temporal consistency. Spatially, meteorological data were assigned to corresponding counties based on the spatial distribution of meteorological stations within administrative boundaries. All datasets underwent rigorous quality control procedures prior to analysis.

### 2.3 Statistical analyses

#### 2.3.1 Spatial and ecological analyses

Spatial visualization was conducted at two scales. At the county level, mosquito density was log-transformed using log_10_(x + 1) prior to mapping to reduce skewness. At a finer scale, inverse distance weighting (IDW) interpolation based on sampling locations was used to generate continuous spatial distribution surfaces. Urban–rural differences in mosquito density were assessed using the Wilcoxon signed-rank test. Mosquito community composition was evaluated using non-metric multidimensional scaling (NMDS) based on Bray-Curtis dissimilarity, with group differences tested using permutational multivariate analysis of variance (PERMANOVA). Similarity percentage (SIMPER) analysis was performed to identify taxa contributing to community dissimilarities. Spearman’s rank correlation analysis was performed to assess associations between mosquito density and meteorological variables. To characterize antecedent meteorological conditions, we calculated moving-average exposures for temperature, relative humidity, and wind speed and cumulative exposures for rainfall and sunshine duration over prespecified lag windows. For example, Temp_MA1–7 denotes the mean daily temperature over lag days 1–7 before each mosquito collection, and Sun_Cum1–7 denotes the cumulative sunshine duration over the same lag window. The collection day was not included in these windows.

#### 2.3.2 GAM-based exposure–response and threshold analysis

A generalized additive model (GAM) was used to examine nonlinear associations between meteorological variables and log_10_-transformed mosquito density. Candidate lag-window models were compared using the Akaike information criterion (AIC), and the final model was fitted using REML in the mgcv package. Exposure–response curves were evaluated over the 5th–95th percentile range, with other meteorological covariates and time fixed at their median values and the district-level random effect excluded. First derivatives of the smooth functions were estimated using central finite differences in the gratia package. Simultaneous 95% confidence intervals were calculated using 10,000 simulations, and potential thresholds were identified from sustained significant derivative intervals and derivative zero crossings.

#### 2.3.3 Predictive modeling and joint-response analysis

The data were divided into training (80%) and test (20%) sets for model development and performance evaluation. Observations collected on the same date were assigned to the same partition to reduce information leakage. Lagged predictors were selected exclusively within the training set by comparing maximum-likelihood GAMs using AIC. GAM, random forest, and XGBoost models were then developed using an identical predictor set. The GAM was fitted using REML, whereas RF and XGBoost hyperparameters were optimized using five-fold date-grouped cross-validation within the training set. Predictive performance was evaluated in the held-out test set using predictive R², RMSE, MAE, and bias. Uncertainty in performance estimates was assessed using 2,000 date-clustered bootstrap replicates. Temperature–sunshine joint-response surfaces were evaluated over the 5th–95th percentile ranges of the training exposures. Other meteorological variables and time were fixed at their median values. County-specific random effects were excluded from GAM predictions, whereas RF and XGBoost predictions were averaged to obtain comparable overall response estimates. Additive and tensor-product GAMs were compared using AIC, and cross-model disagreement was quantified as the pointwise standard deviation of the estimated non-additive interaction components. All analyses described above were performed in R (v4.2.2; http://cran.r-project.org/).

## 3 Results

### 3.1 Spatial distribution and composition of mosquito populations

The spatial distribution of mosquito density showed significant heterogeneity. Overall, lower mosquito densities were observed in central urban districts, including Jinjiang, Chenghua, Longquanyi, and Tianfu New Area. In contrast, higher values were predominantly detected in peripheral regions, such as Chongzhou (2.66), Dujiangyan (2.01), Qionglai (1.21), and Jintang (1.15). Intermediate mosquito densities were observed in transitional areas, including Wenjiang, Pidu, Xindu, and Qingbaijiang (**Figure 1A**).

**Figure 1:**
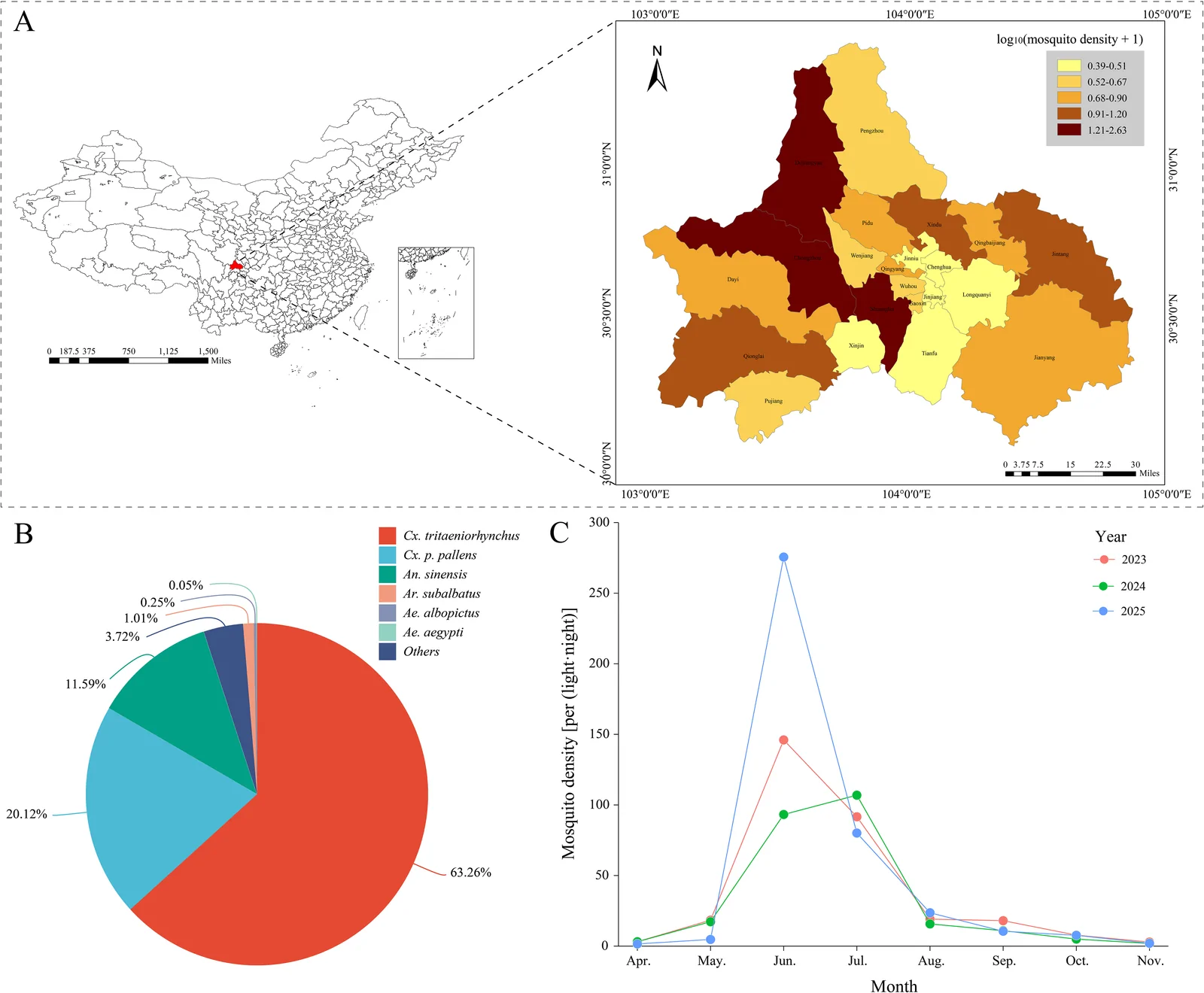
Spatiotemporal variation and species composition of mosquitoes in Chengdu from 2023 to 2025. **(A)** Spatial distribution of mosquito density at the county level. Mosquito density was log_10_(x + 1)-transformed for visualization. **(B)** Species composition of mosquitoes. **(C)** The seasonal distribution of mosquito density in Chengdu from 2023 to 2025.

Between 2023 and 2025, a total of 5,556 mosquito traps were deployed, capturing 224,996 female mosquitoes. The collected specimens included *Culex tritaeniorhynchus* (142,333, 63.26%), *Cx. pipiens pallens* (45,270, 20.12%), *Anopheles sinensis* (26,074, 11.59%), *Armigeres subalbatus* (2,278, 1.01%), *Aedes albopictus* (558, 0.25%), and *Ae. aegypti* (102, 0.05%) (**Figure 1B**). Species composition differed significantly among habitats (*χ*² = 44775, *P* < 0.001), with pasture sheds exhibiting the highest average mosquito density of 241.17 per light-trap night, which was substantially higher than other habitats (**Table 1**). In pasture sheds, *Cx. tritaeniorhynchus* exhibited the highest proportion (68.62%), whereas *Cx. p. pallens* was the dominant species in other habitats. The mosquito density exhibited a unimodal curve, peaking at 146.0, 106.9, and 275.6 per light-trap night in June 2023, July 2024, and June 2025, respectively (**Figure 1C**). Across the observed year-specific curves, the maximum recorded density occurred in June for *Cx. tritaeniorhynchus*, *An. sinensis*, and *Ae. aegypti*; in July for *Cx. p. pallens*; in August for *Ae. albopictus*; and in September for *Ar. subalbatus* (**Supplementary Figure 1**).

**Table 1:** The proportional composition of mosquito species in different habitats in Chengdu from 2023 to 2025.

Habitat/species	*Cx. p. pallens*	*Cx. tritaeniorhynchus*	*Ae. albopictus*	*Ae. aegypti*	*An. sinensis*	*Ar. subalbatus*	*Others*
Residential areas	76.46%	12.22%	3.24%	0	1.18%	1.41%	5.48%
Parks	79.03%	9.40%	4.42%	0	0.80%	1.48%	4.87%
Hospitals	81.65%	5.35%	2.87%	0	1.15%	1.29%	7.69%
Farms	49.54%	32.38%	0.56%	0.03%	5.42%	2.40%	9.67%
Pasture sheds	14.64%	68.62%	0.05%	0.05%	12.65%	0.87%	3.12%

### 3.2 Urban-rural differences in mosquito density, spatial distribution, and community structure

Residential areas, hospitals, and parks, characterized by a high degree of urbanization and similar levels of anthropogenic disturbance, were defined as urban habitats, whereas farms and pastures were defined as rural habitats. The Wilcoxon signed-rank test revealed a significant difference in mosquito density between urban and rural habitats (*P* < 0.001) (**Figure 2A**). Spatial interpolation revealed multiple high-density areas around rural sampling sites, whereas urban areas exhibited lower mosquito densities and a relatively homogeneous spatial distribution (**Figure 2B**). In addition to differences in mosquito density, the mosquito community structure in urban and rural areas was distinctly separated (stress = 0.101). PERMANOVA analysis further revealed that habitat type had a significant effect on mosquito community composition (*R*² = 0.537, *P* = 0.001) (**Figure 2C**). The community differences were primarily driven by a few dominant species. *Cx. p. pallens* and *Cx. tritaeniorhynchus* contributed the most to the differences, together explaining most of the dissimilarities between the communities. In terms of habitat preference, *Cx. p. pallens* and *Ae. albopictus* were relatively more abundant in urban habitats, while *Cx. tritaeniorhynchus, Ar. subalbatus*, and *An. sinensis* had higher relative abundances in rural habitats (**Figure 2D**).

**Figure 2:**
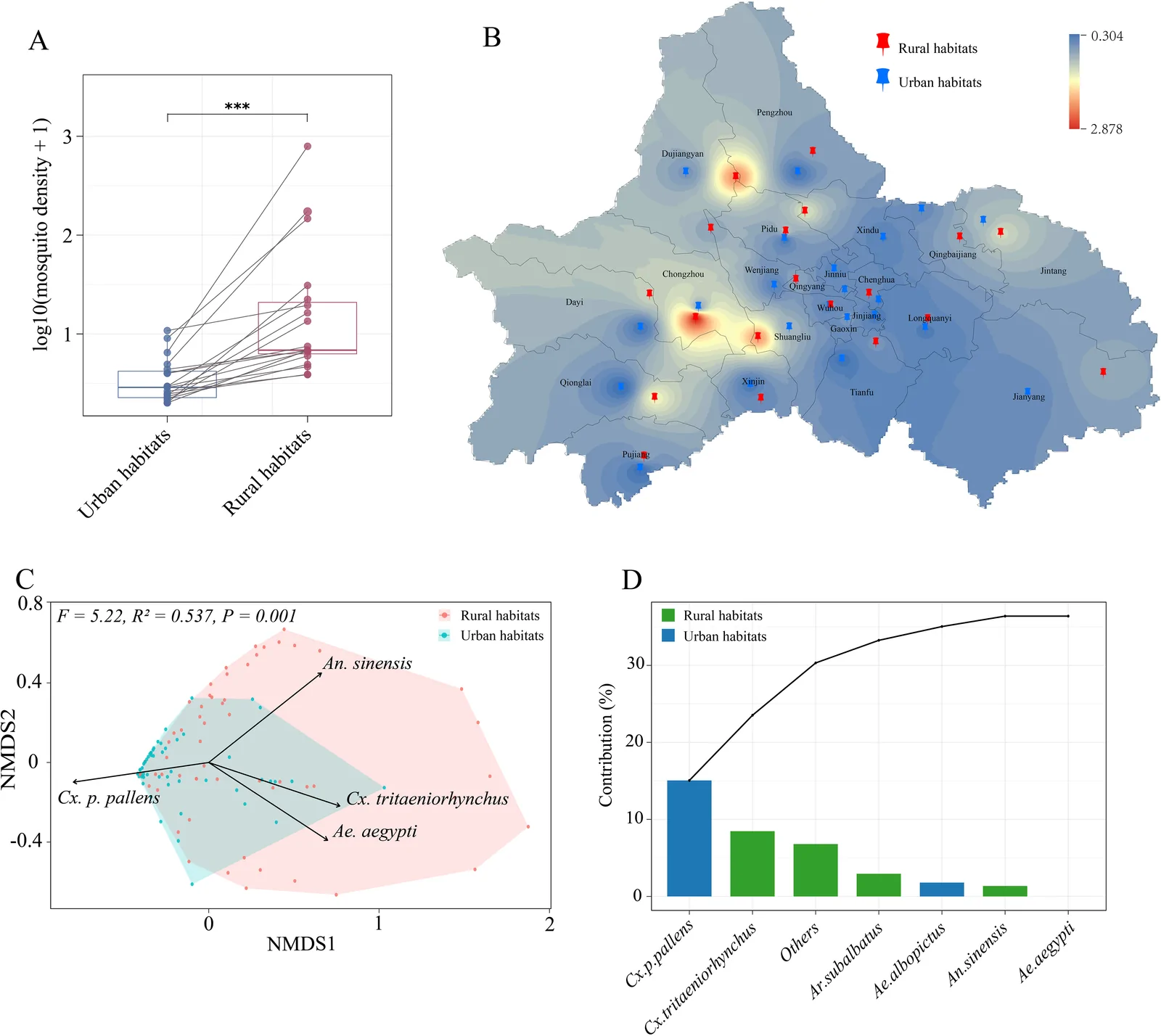
Differences in mosquito density, spatial distribution, and community composition between urban and rural habitats. **(A)** Paired comparison of mosquito densities between urban and rural habitats (***P < 0.001). **(B)** Spatial distribution of mosquito density based on IDW interpolation. **(C)** NMDS ordination of mosquito communities in different habitats, with polygons representing habitat groupings and group differences were assessed using PERMANOVA. **(D)** Species contributions to community dissimilarity between habitats based on SIMPER analysis. Bars indicate individual species contributions, and the line represents the cumulative contribution.

### 3.3 Meteorological correlations of mosquito density: lagged associations and nonlinear responses

To ensure the spatial correspondence between meteorological and mosquito-density data, subsequent analyses were restricted to nine counties, each with at least one meteorological monitoring station located within its administrative boundaries. The correlation analyses revealed significant positive correlations between temperature and mosquito density across multiple lag windows (**Figure 3**).

**Figure 3:**
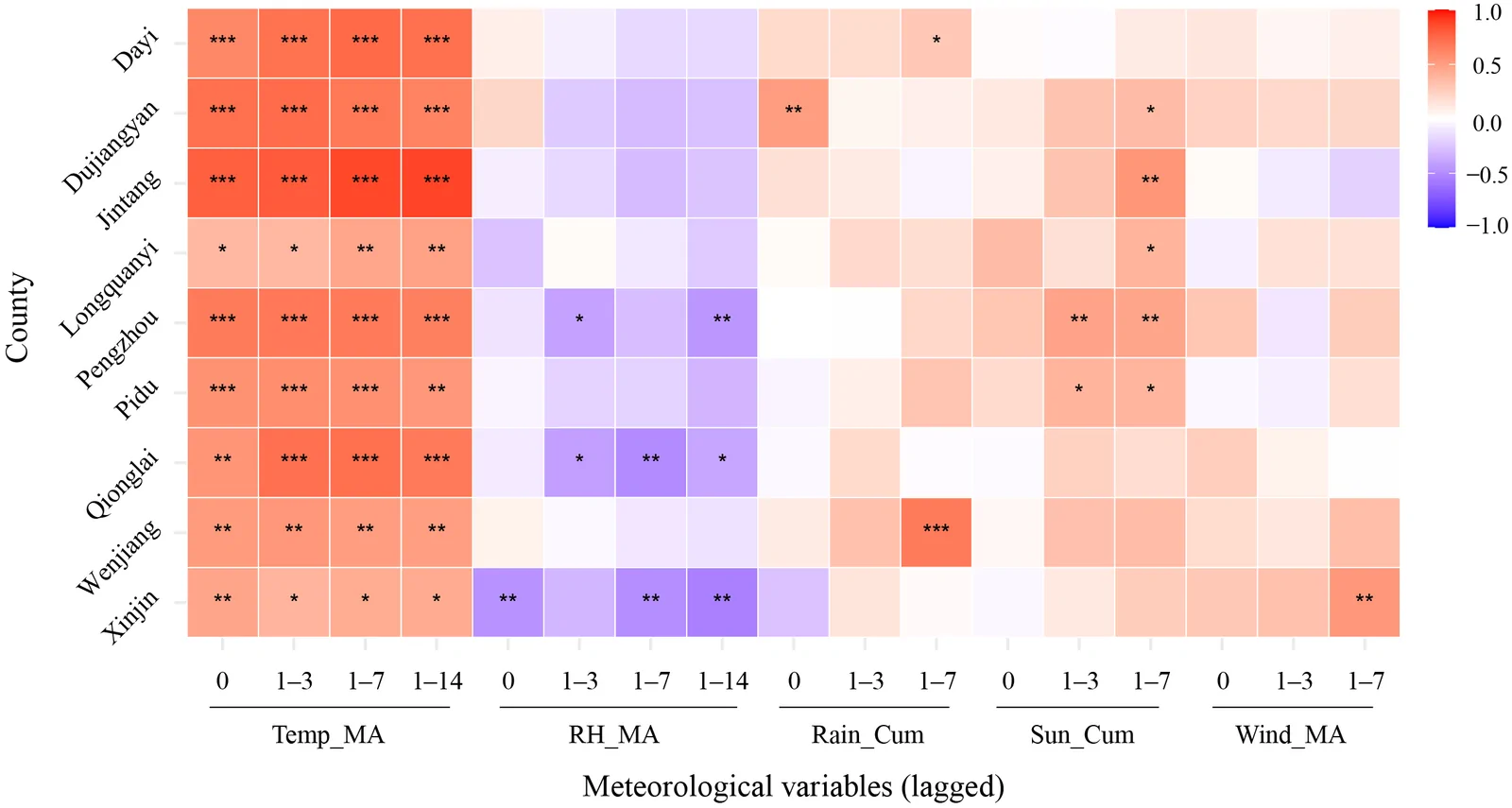
Correlations between mosquito density and lagged meteorological variables. Color gradients represent Spearman correlation coefficients (ρ), and statistical significance is indicated as *P < 0.05, **P < 0.01, and ***P < 0.001. Temp, temperature; RH, relative humidity; Rain, rainfall; Sun, sunshine duration; Wind, wind speed.

The GAM was further fitted to quantify nonlinear associations between lag-window aggregated meteorological variables and mosquito density. Temporal terms were incorporated to control for long-term and seasonal trends, and administrative regions of Chengdu were included as random effects to account for potential spatial heterogeneity. The results indicated that mean daily temperature over lag days 1–7 (Temp_MA1–7) was the primary significant influencing factor (Edf = 4.267, *F* = 25.152, *P* < 0.001), while cumulative sunshine duration over lag days 1–7 (Sun_Cum1–7) also showed a statistically significant effect (Edf = 3.587, *F* = 4.572, *P* < 0.001) (**Table 2**). Based on the derivative analysis of the GAM smooth, mosquito density began to increase significantly at Temp_MA1–7 of 15.59 °C and reached peak at 25.02 °C, followed by a gradual but non-significant decline (**Figures 4A, B**). For Sun_Cum1–7, mosquito density reached a local minimum at 38.0 h and subsequently increased, with a significant increase at 52.94 h (**Figures 4C, D**). Relative humidity (MA1–14), rainfall (Cum1–7), and wind speed (MA1–7) were not significantly associated with mosquito density (all *P* > 0.05). The random effects of counties were significant (Edf = 7.816, *F* = 47.760, *P* < 0.001), whereas the temporal trend term was not significant (*P* > 0.05).

**Table 2:** GAM estimates for associations between lagged meteorological predictors and mosquito density in Chengdu from 2023 to 2025.

Variables	Edf	*F/t*	*P*
Intercept	–	6.068	<0.001
Mean daily temperature over lag days 1–7	4.267	25.152	<0.001
Mean daily relative humidity over lag days 1–14	1.000	0.042	0.839
Cumulative rainfall over lag days 1–7	1.620	0.150	0.833
Cumulative sunshine duration over lag days 1–7	3.587	4.572	<0.001
Mean daily wind speed over lag days 1–7	1.582	1.277	0.277
Time	<0.001	<0.001	0.593
District (random effect)	7.816	47.760	<0.001

**Figure 4:**
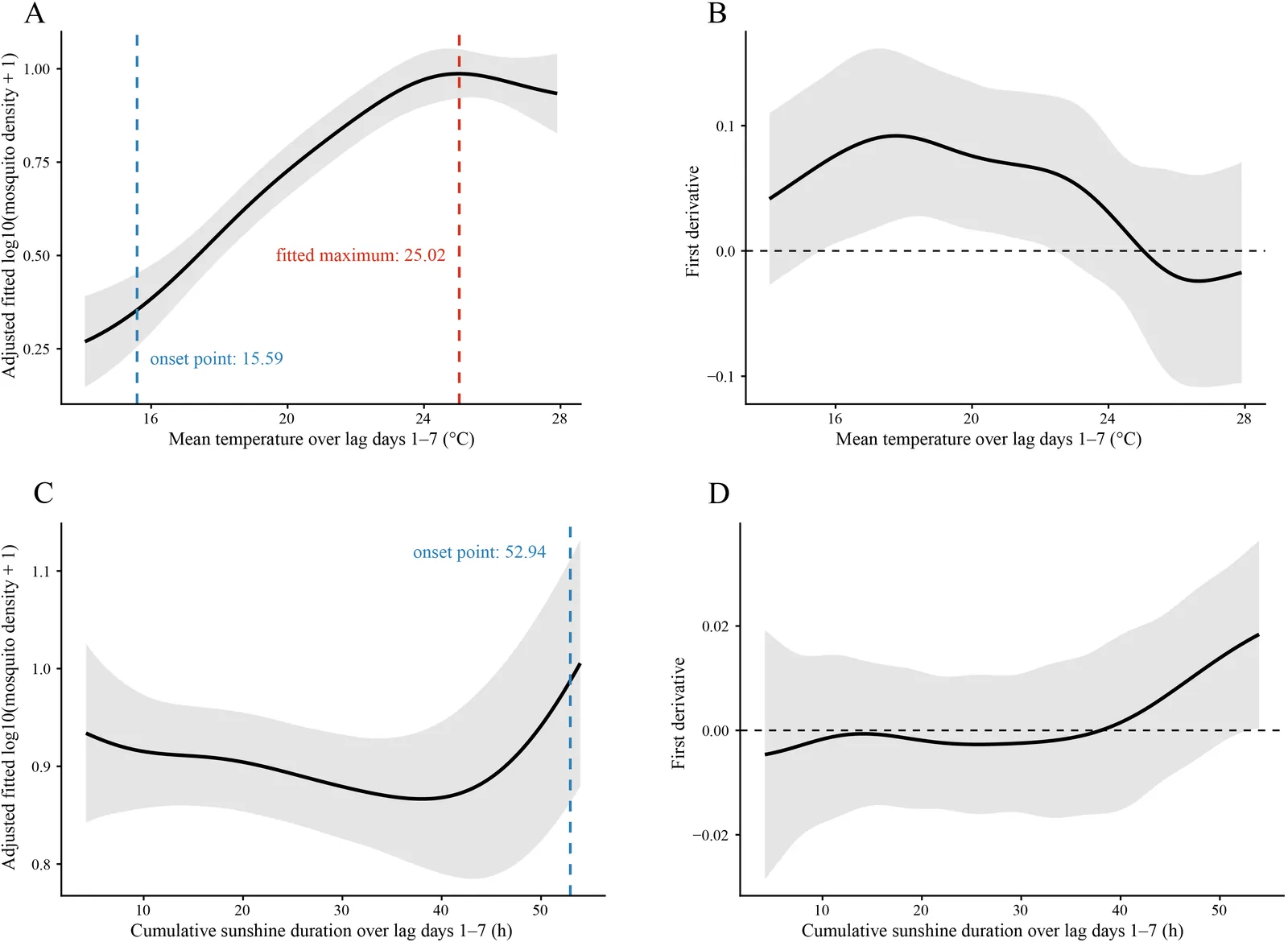
Adjusted exposure–response relationships between mosquito density and meteorological exposures over lag days 1–7 and their corresponding first derivatives. **(A, C)** Adjusted exposure–response curves for temperature and sunshine duration, respectively. **(B, D)** Corresponding first derivatives. Shaded areas denote simultaneous 95% confidence intervals; blue dashed lines indicate derivative-based onset points, whereas the red dashed line indicates the fitted maximum.

### 3.4 Model performance and temperature–sunshine joint response patterns

Using the same set of meteorological predictors, we trained GAM, RF, and XGBoost models on the training dates and evaluated their out-of-sample predictive performance on the held-out dates. On the held-out dates, RF achieved the highest predictive *R²* (0.715), followed by XGBoost (0.642) and GAM (0.609) (**Figure 5A**). In addition, RF showed the lowest prediction errors (RMSE = 0.281, MAE = 0.220), followed by XGBoost (RMSE = 0.315, MAE = 0.252), whereas GAM exhibited higher errors (RMSE = 0.329, MAE = 0.260). All models showed positive bias, with GAM exhibiting the largest deviation (0.022). The two-dimensional joint-response surfaces for temperature and sunshine duration showed GAM produced a smooth, temperature-dominated gradient (**Figure 5B**), while RF and XGBoost generated more stepwise surfaces (**Figures 5C, D**). Despite local differences, the overall joint-response patterns were broadly consistent across models, with greater agreement at mean temperatures of 18–22 °C and cumulative sunshine durations of 10–45 h over lag days 1–7 (**Figure 5E**).

**Figure 5:**
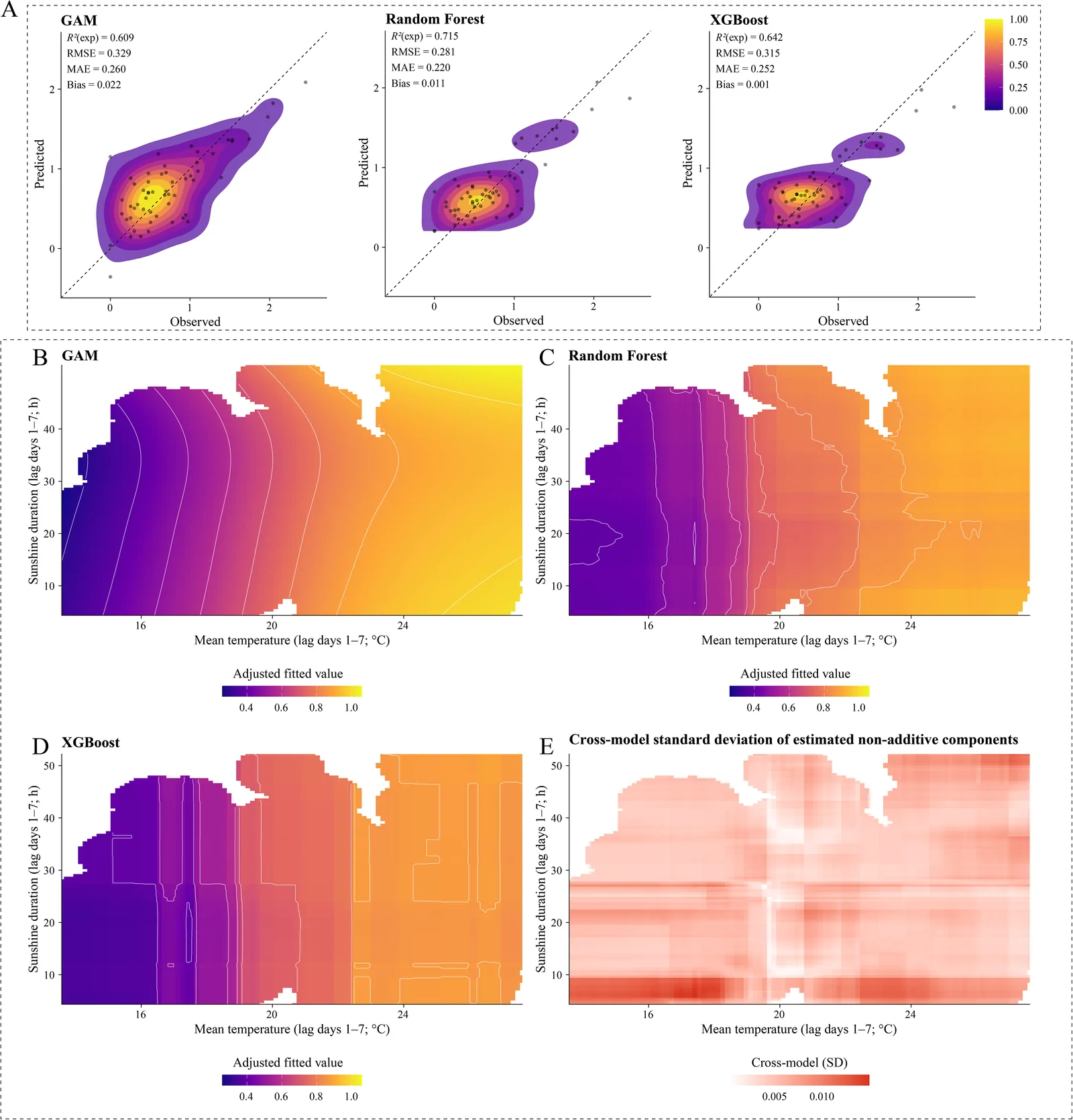
Predictive performance and joint temperature–sunshine response patterns of GAM, RF, and XGBoost models. **(A)** Observed versus predicted log_10_-transformed mosquito density in the held-out test data; colors indicate relative point density, and the dashed line is the 1:1 reference line. **(B–D)** Joint response surfaces estimated by GAM, RF, and XGBoost, respectively. **(E)** Cross-model standard deviation of the estimated non-additive interaction components.

## 4 Discussion

This study investigated mosquito density and species distribution in Chengdu from 2023 to 2025, as well as the effects of meteorological factors on mosquito density. The results showed that mosquito density exhibited significant spatial heterogeneity, with a clear spatial gradient characterized by lower values in the central urban area and higher values in peripheral regions. This spatial pattern was closely associated with the urbanization gradient and differences in ecological environments. Specifically, Jinjiang, Chenghua, Jinniu Districts, and Tianfu New Area were characterized by high levels of urbanization, a high proportion of impervious surfaces, and strong anthropogenic disturbance. In contrast, peripheral areas such as Chongzhou, Dujiangyan, and Qionglai contained more abundant water bodies, farmland, and semi-natural habitats, providing more suitable conditions for mosquito habitation and reproduction (Dong et al., 2022; Huang and Yang, 2025).

The comparison between urban and rural areas further revealed generally higher mosquito densities in rural habitats. In addition, community structure analysis showed that the composition of mosquito communities in urban and rural areas was significantly separated, indicating that not only were there differences in mosquito abundance, but also in species composition across habitats. This community differentiation was primarily driven by a few dominant species, with *Cx. p. pallens* and *Cx. tritaeniorhynchus* contributing the most, suggesting that ecological differences in adaptability among dominant species may play an important role in shaping community structure (Juliano, 2009; Laporta and Sallum, 2014). *Cx. tritaeniorhynchus* and *Cx. p. pallens* were the dominant species in Chengdu, accounting for 83.38% of the total mosquitoes captured by light traps, consistent with surveillance results from other regions in China. Notably, mosquito density in pasture sheds habitats was significantly higher than in other habitats, with a mean of 241.17 per light-night. In this habitat, *Cx. tritaeniorhynchus* showed the highest proportion, which may be related to its zoophilic preference and the environmental characteristics of pasture areas, including high organic matter availability, abundant animal hosts, and relatively stable microclimatic conditions that may provide favorable conditions for breeding and blood-feeding. This finding highlights the importance of human-livestock interfaces in shaping mosquito-borne disease transmission risk, particularly for Japanese encephalitis, where *Cx. tritaeniorhynchus* serves as the primary vector (McGuinness et al., 2023; Levesque et al., 2024). In contrast, urban environments are more suitable for the survival of *Ae. albopictus*, which is associated with its adaptability to small water containers, artificial habitats, and the urban heat island effect (Ravasi et al., 2022; Montgomery et al., 2025). *Cx. pipiens pallens*, a member of the *Cx. pipiens* complex widely distributed in northern China, was widely distributed in both urban and rural areas in this study, suggesting strong ecological adaptability and environmental tolerance (Haba and McBride, 2022). Potential interspecific competition may also contribute to the structuring of mosquito communities in urban habitats. In this study, *Cx. p. pallens* and *Ae. albopictus* were relatively more abundant in urban habitats, indicating that they can coexist within the same broad urban environment. Where their larval habitats overlap, competition for limited food and space could affect larval survival and subsequent adult abundance (Carrieri et al., 2003; Juliano, 2009). Nevertheless, these species may exploit partly different aquatic microhabitats. Moreover, their observed density peaks occurred in different months—July for *Cx. p. pallens* and August for *Ae. albopictus*. Such microhabitat and temporal partitioning may reduce direct competition and facilitate coexistence (Laporta and Sallum, 2014; Marini et al., 2017). It should be noted that the urban–rural classification in this study was based on habitat type rather than a continuous measure of urbanization and therefore primarily reflects differences between sampled habitat contexts. In addition, the exclusive use of light traps may have introduced species-specific sampling bias and did not allow direct assessment of larval co-occurrence or resource competition (Hou et al., 2021). Future studies incorporating elevation, population density, vegetation cover, and water cover, together with complementary adult and larval sampling methods, could better distinguish the roles of environmental conditions, niche partitioning, and interspecific competition in shaping mosquito spatial distribution and community composition. Overall, although mosquito density was higher in rural habitats, the risk of mosquito-borne disease transmission is jointly determined by mosquito abundance, dominant species composition, and human exposure (de Souza and Weaver, 2024). The urban-rural transition zone may represent an area that may warrant enhanced vector surveillance, as it combines elevated mosquito density with frequent human exposure. In addition, the coexistence of different ecological groups of species (e.g., *Cx. tritaeniorhynchus* and *Ae. albopictus*) may further increase transmission complexity.

In recent years, the effects of meteorological factors on mosquito population dynamics have been widely studied (Liu et al., 2023; Abbasi, 2025). Meanwhile, machine learning methods, with strengths in capturing nonlinear relationships and improving predictive accuracy, have been increasingly applied to mosquito density prediction alongside traditional statistical models to elucidate driving mechanisms (Guang et al., 2025; Nair et al., 2025). Studies have shown that the optimal developmental temperature for mosquitoes is 20–30 °C, with peak developmental rates occurring at approximately 25 °C (Rueda et al., 1990; Grech et al., 2015). Below 15 °C, the foraging and biting behavior of adult mosquitoes significantly decreases, larval development is notably delayed, and survival rates decline (Moser et al., 2023). Our study showed that mosquito density increased sharply when mean daily temperature over lag days 1–7 exceeded 15.59 °C, peaked at around 25.02 °C, and subsequently declined, consistent with previous evidence. As temperature increased, the mosquito development period shortened, accelerating egg hatching and reducing the time to emergence, thereby leading to increased mosquito density (Sukiato et al., 2019). However, extreme summer temperatures not only accelerated the aging of the reproductive system, leading to reduced fecundity, but also impaired immune function by decreasing antibacterial activity, thereby reducing mosquito density (Agyekum et al., 2022; Martin et al., 2024, 2025).

Sunshine duration was also significantly associated with mosquito density. Our results showed that when the cumulative sunshine duration over lag days 1–7 exceeded 38 h, mosquito density increased, although a statistically significant positive change was observed only above 52.94 h. The mechanisms underlying the effects of sunshine duration remain uncertain. Some studies suggested that sunshine duration could directly influence mosquito populations by regulating adult diapause and reproductive behavior through photoperiodic signals (Peffers et al., 2021). Other studies have suggested that sunshine duration may affect mosquito density by modifying local temperature and other microenvironmental conditions or by interacting with temperature (Ravasi et al., 2022; Teixeira et al., 2026). Our results showed that in the GAM model, the response of mosquito density to sunshine duration was relatively weak, whereas temperature was the predominant driver. The temperature–sunshine joint response surfaces also showed that mosquito density mainly increased with temperature, with comparatively limited variation along the sunshine axis. The non-additive interaction components identified by RF and XGBoost were also small and did not show a stable synergistic enhancement between temperature and sunshine duration. Therefore, we speculated that the association between sunshine duration and mosquito density may primarily represent an additive effect rather than a stable temperature-dependent synergistic interaction.

Relative humidity, rainfall, and wind speed were not significantly associated with mosquito density in the fitted model, which may be attributed to the relatively homogeneous humidity conditions and limited environmental variability. Moreover, the county-level random effect was significant, indicating substantial spatial heterogeneity among administrative areas that was not explained by the environmental variables included in the model and may reflect unmeasured differences in land-use patterns, microclimatic conditions, and the intensity of human activity. Overall, our findings indicate that mosquito density was primarily associated with temperature. When the short-term mean temperature approached 25 °C, particularly in administrative areas with persistently high mosquito densities, mosquito surveillance, breeding site management, and adult mosquito control could be strengthened to improve the timeliness and targeting of interventions.

In terms of predictive performance, RF performed best in predicting mosquito density, followed by XGBoost, whereas GAM showed comparatively lower predictive accuracy. The error metrics (RMSE and MAE) also supported this trend, indicating that tree-based ensemble learning methods had a stronger ability to capture the complex relationships between mosquito density and meteorological factors. This result was consistent with previous studies, which showed that random forest and gradient boosting models were more effective in handling higher-order interactions and nonlinear relationships among variables (Ong et al., 2023; Ferraguti et al., 2024; Kuo et al., 2024). Although a GAM can model nonlinear relationships, its smoothness constraints may have limited its ability to capture complex local structures (Elith et al., 2006; Elith et al., 2008). In the present study, the overall non-additive temperature–sunshine interaction components were small; therefore, differences in predictive performance cannot be directly interpreted as evidence of higher-order interactions. Overall, mosquito density showed clear nonlinear responses to meteorological factors, whereas evidence for stable interactions remained limited. Future studies should incorporate data from additional years and regions to further investigate coupling mechanisms and threshold effects among multiple meteorological variables.

## 5 Conclusions

Our findings indicated mosquito density in Chengdu showed marked seasonal variation, spatial heterogeneity, and urban–rural differences in community composition. Temperature and sunshine duration were nonlinearly associated with mosquito density over selected 7-day exposure windows. Compared with the GAM model, machine learning models showed superior predictive performance for mosquito density, highlighting their potential for mosquito density prediction under complex meteorological conditions. These findings support incorporating meteorological information, particularly temperature, into mosquito early-warning systems and targeted vector control to reduce potential mosquito-borne disease risks.

## Data Availability

The raw data supporting the conclusions of this article will be made available by the authors, without undue reservation.
