# Supplementary material for: Spatiotemporal patterns and meteorological associations of mosquito density: insights from mosquito surveillance in Chengdu, China
Source: Front Cell Infect Microbiol. 2026 Sep 16;16:1948618. doi: 10.3389/fcimb.2026.1948618 (PMC13623562; doi:10.3389/fcimb.2026.1948618)
Supplement: Supplementary file 1 [file Table1.docx]

Supplementary Material

**Figures**

**Supplementary Figure 1.** Monthly mosquito density by species and year in Chengdu from 2023 to 2025.
